# Extended view on the mechanobiology of fracture healing: interplay between mechanics and inflammation

**DOI:** 10.3389/fbioe.2025.1652897

**Published:** 2025-10-10

**Authors:** Nico Gläser, Maria Schröder, Jan Barcik, Melanie Haffner-Luntzer, Esther Wehrle

**Affiliations:** ^1^ Institute of Orthopaedic Research and Biomechanics, University Medical Center Ulm, Ulm, Germany; ^2^ AO Research Institute Davos, Davos Platz, Switzerland

**Keywords:** fracture, fracture healing, mechanobiolgy, inflammation, bone

## Abstract

It is well established that the biomechanical environment guides bone regeneration. It is also commonly accepted that the early inflammatory phase of fracture healing is decisive for the later regeneration process by inducing angiogenesis, stem cell invasion and cartilage and bone tissue formation. While traditionally, biomechanical orchestration and inflammation were viewed as distinct phenomena, recent research has illuminated the intricate relationship between mechanics and inflammation in the mechanobiology of fracture healing. In this review, we summarize the current knowledge of how mechanical stimuli influence bone regeneration by inducing tissue differentiation, and we broaden the perspective on the mechanobiology of fracture healing by incorporating recent insights into the interaction between mechanical forces and inflammation—an emerging field termed as “mechano-immunomics.” Key topics include the impact of fixation stiffness on immune cell migration and early gene expression of extracellular matrix-modulating genes, the influence of the mechanical environment within the early fracture hematoma on platelets and immune cells, and whether external biomechanical stimulation can alter the mechano-immunomic landscape. Gaining a deeper understanding of this dynamic interplay offers promising opportunities for innovative therapeutic strategies to enhance fracture healing. However, significant challenges remain, such as the development of suitable *in vitro* systems, well-characterized *in vivo* models, and effective interdisciplinary collaboration across the fields of biology, immunology, and biomechanics.

## 1 Introduction

Bone healing is a complex biological process involving the tight interaction between many different cell types with complete restoration of the natural structure, shape and mechanical competence of the bone. While most bone fractures heal uneventfully, 5%–10% of all fractures are described to be of risk for a delayed healing or non-union formation (Bahney et al., 2019; Hellwinkel et al., 2020). The reasons are not fully known, although it is evident from clinical practice that the biomechanical environment at the fracture site is critical for successful healing. Decades of basic, translational and clinical research focused on the orchestration of callus development and bone healing by mechanical forces (Claes, 2021). In recent years, it became more and more evident that also the inflammatory response of the bone to a fracture is decisive for the later regeneration (Duda et al., 2023). While traditionally biomechanical orchestration and inflammation were viewed as distinct phenomena, recent research has illuminated the intricate relationship between mechanics and inflammation in the mechanobiology of fracture healing (Schmidt-Bleek et al., 2014). This extended perspective narrative review article sheds light on how mechanical cues influence the inflammatory milieu during the healing process. Understanding this dynamic interplay holds promise for innovative therapeutic interventions aimed at optimizing the settings for fracture repair and structural healing outcomes. We performed a PubMed-based literature research using the terms “mechanobiology”/“mechanotransduction”/“mechanosensation”/“biomechanics”/“biomechanical stimulation”/“biomechanical simulation” together with the terms “fracture healing”/“bone regeneration”/“bone healing” and further “inflammation”/“inflammatory”/“immune cells”. We included publications relevant for the topic of mechanobiology during fracture healing in the context of this review. We did not perform a systematic review approach for this manuscript.

## 2 Fracture healing process

Fracture healing is a complex and dynamic regenerative process completely restoring the damaged bone to its pre-injury structure. The whole process and the underlying molecular mechanisms have been investigated using preclinical large- and small-animal models (Haffner-L et al., 2020). Various cell types, both from the hematopoietic and the mesenchymal lineage, in company with their secreted factors, are important for the regeneration process. In addition to these biological factors, bone regeneration is also dependent on mechanical strains and stresses at the fracture site. The fracture healing process can be divided in three different phases which should not be regarded as separated processes and rather partly overlap and influence each other: the inflammatory phase, the repair phase, and the remodeling phase (Bahney et al., 2019).

### 2.1 Inflammatory phase

The whole process starts with the inflammatory phase immediately after the fracture when disruption of blood vessels happens with a subsequent formation of a fibrin-rich blood clot around the fractured bone ends (Hu et al., 2017). This early fracture hematoma is characterized by low oxygen levels and decreased tissue pH because of the limited oxygen supply in the injured area (Lu et al., 2011). The injury further induces the release of damage-associated molecular patterns (DAMPs) from damaged cells, supplemented by pathogen-associated molecular patterns (PAMPs) in the case of an open fracture event (Kovtun et al., 2016). In addition, platelets arrive at the fracture site and become activated. These activated non-nucleated cells secrete different cytokines such as interleukin (IL)-1, IL-6 and tumor necrosis factor-alpha (TNF- α ) (Baht et al., 2018; Jingushi et al., 1995; Dimitriou et al., 2005; Dulgeroglu and Metineren, 2017). Further, growth factors such as platelet-derived growth factor (PDGF), vascular endothelial growth factor (VEGF), Insuline-like growth factor 1 (IGF-1), and transforming growth factor-beta (TGF-ß) are released. Various immune cells are recruited to the injured area by these signaling molecules (Gibon et al., 2017). The first immune cells rapidly recruited to the early fracture hematoma are polymorphonuclear neutrophils (PMNs), which facilitate the removal of cell debris and pathogens through processes like phagocytosis, the formation of neutrophil extracellular traps (NETs) (Kovtun et al., 2016; Kolaczkowska and Kubes, 2013) and secretion of inflammatory mediators (Kovtun et al., 2018). C-C motif chemokine ligand 2 (CCL2 and its receptor chemokine receptor type 2 (CCR2) play a pivotal role in mediating subsequent monocyte chemotaxis (Chu et al., 2014). Monocytes differentiate into pro-inflammatory M1 macrophages, contributing to the innate immune response to PAMPs through toll-like receptors (TLRs) (Claes et al., 2012; Einhorn and Gerstenfeld, 2015). Following wound debridement and the cessation of classical activation, macrophages can adopt an anti-inflammatory M2 state through IL-4 and IL-13 signaling (Alexander et al., 2011; Vi et al., 2015). A recent study from Zou et al. showed higher secretion of prosenescent factors as grancalcin by macrophages in calluses during aging which led to senescence of skeletal stem/progenitor cells (SSPS). This mechanism results in an impaired fracture healing by reducing the regeneration capability of the bone (Zou et al., 2024). Moreover, studies also investigated the role of mast cells in bone fracture healing. They showed that the increased systemic posttraumatic inflammation after severe trauma and the dysregulated early immune response at the fracture site in case of an additional trauma are related to mast cells. One of the key cytokines of posttraumatic inflammation is IL-6 which is significantly reduced in mast cell-deficient mice 3 hours after fracture compared to wildtype, indicating that mast-cell derived mediators might play a role in fracture healing (Fischer et al., 2022; Ragipoglu et al., 2022; Ragipoglu et al., 2020; Fischer and Haffner-Luntzer, 2022). Also, cells from the adaptive immune system, such as T lymphocytes, are recruited to the fracture site as well (Konnecke et al., 2014). They have also been demonstrated to play a role in bone healing. Notably, RAG1^−/−^ mice, characterized by a complete absence of the adaptive immune system, exhibited increased callus mineralization, primarily attributed to the absence of T lymphocytes (El Khassawna et al., 2017)^,^ (Reinke et al., 2013). T cell response is strongly controlled by RANKL-activated osteoclasts as they have been shown to activate T cells (Amarasekara et al., 2018; Kiesel et al., 2009). In addition to inflammatory and anti-inflammatory cytokines, angiogenic factors such as angiopoetin-1 and VEGF are released (Ai-Aql et al., 2008). This initiates the migration of endothelial cells from pre-existing periosteal vessels towards the fracture hematoma, facilitating the formation of new blood vessels crucial for revascularization during fracture healing (Schmidt-Bleek et al., 2012). These newly formed blood vessels play a pivotal role in enabling the influx of osteoprogenitor cells and fibroblasts, which contribute to the production of new collagen. This collagen-rich granulation tissue replaces the hematoma and is characterized by the presence of cells and invading capillaries (McKibbin, 1978). All these processes and the numerous cell types, mediators and pathways underline the importance of the inflammatory phase after bone fracture on ongoing healing and regeneration processes.

### 2.2 Repair phase

Dependent on the local mechanical conditions in the fracture area, intramembranous and endochondral ossification processes take place during the repair phase of fracture healing. Primary cortical bone healing is characterized by intramembranous bone healing via Haversian remodeling, similar to the normal bone remodeling facilitated by direct contact of the fracture surfaces and high oxygen supply (Holzwarth et al., 2010). Osteoclasts, establish resorbed tunnels from one side of the fracture to the other. Subsequently, blood vessels can proliferate within these tunnels what enables the recruitment of precursor cells that undergo differentiation into bone-forming osteoblasts (Einhorn, 2005). These osteoblasts generate new osteons to bridge the fragments of the fracture, marking a gradual process with a reduced influx of inflammatory cells, potentially reducing systemic inflammation. Primary bone healing is mainly applied in trauma surgery using compression plates (Bastian et al., 2011). Secondary bone healing occurs under non-rigid fixation via the processes of intramembranous and endochondral ossification. Intramembranous ossification takes place distal to the fracture site, along the periosteal and endosteal bone surfaces, involving direct bone formation by osteoblasts derived from osteoprogenitor cells in the periosteum. Conversely, bone formation originating from a soft fracture callus occurs between and around the fracture ends. Mesenchymal stem cells (MSCs) from the bone marrow and periosteum are recruited to the fracture site, where they differentiate into chondrocytes (Bahney et al., 2019). Chondrogenesis is subsequently induced by high tissue strains and reduced oxygen saturation in the vicinity of the fracture gap (Claes et al., 2011a). Once the cartilaginous callus has bridged the fracture and interfragmentary movements decrease thereby, chondrocytes undergo hypertrophy and promote vascularization by expressing factors like VEGF (Bahney et al., 2019). Subsequently, the monocytes differentiate into cartilage-resorbing osteoclasts, while the invaded MSCs differentiate into osteoblasts, contributing to a bony bridging by filling the resorption lacunae of the callus with woven bone. Osteoblasts transdifferentiated from chondrocytes also play a role in the bony bridging of the fracture callus, as evidenced by studies using lineage tracing mouse models (Hu et al., 2017; Zhou et al., 2014).

### 2.3 Remodeling phase

The bone remodeling phase may persist for months to years following the clinical union of the fracture. Throughout this remodeling phase, various signaling pathways, including BMP, fibroblast growth factor (FGF), parathyroid hormone-related peptide (PTHrP), and Indian hedgehog (IHH), play a role (Sheen et al., 2024). During the remodeling phase, a balance between osteoblasts and osteoclasts, which are highly present in this phase is necessary. Especially the activity and differentiation of osteoclasts is enhanced by pro-inflammatory cytokines as TNF-alpha, IL-1, IL-6, IL-11 and IL-17, which are also shown to inhibit osteoblast differentiation, function and collagen synthesis and which can additionally be produced by macrophages (Lorenzo, 2000; Gilbert et al., 2000; Cavagis et al., 2014). Bone remodeling is also heavily influenced by mast cells. For instance, Ragipoglu et al. could show significantly reduced osteoclast numbers in mast cell-deficient mice 21 days after fracture and an impaired callus remodeling (Ragipoglu et al., 2022). The woven bone within the callus undergoes conversion into lamellar bone by osteon formation and the vascularization is reduced to pre-fracture levels. The bone remodeling phase can take years in humans but ends with the restoration of the original bone architecture as the final target of the fracture healing process (Bahney et al., 2019).

## 3 “Mechanobiology–state-of-the-art”: role of the biomechanical environment during fracture healing

### 3.1 Clinical experience on healing mechanics

During the healing process, the fracture callus stabilizes fractured bone fragments and later unites them to achieve pre-injury strength and stiffness of the broken bone. Therefore the process of callus formation is, and must be, sensitive to the local mechanical conditions that act in the fracture gap (Epari et al., 2007; Yamagishi and Yoshimura, 1955). In clinical settings, these local mechanical conditions result from the interplay between fracture geometry, fixation stiffness and the physiological loading of the broken bone. When a patient loads the operated limb, the implant deforms, causing movements of bone fragments with respect to each other that, in turn, strain the tissue in the fracture gap and stimulates fracture healing (Inacio et al., 2023; Claes, 2011; Duda et al., 2002). Over the last century, clinical experience and research on the mechanobiology of bone fracture healing has changed the paradigm of how bone fractures should be treated. Open-reduction techniques—targeted towards perfectly restoring bone continuity and geometry to allow for primary bone healing—were (whenever possible) replaced with 1) minimally invasive approaches for prevention of extensive damage of soft tissue around the fracture, thereby accounting for enhanced biology at the fracture site (Perren, 2002; Windolf et al., 2023; Thaeter et al., 2016) and 2) fixation techniques which stimulate fracture callus formation. This was done by introducing flexible internal (or external) fixators (Perren, 2002; Schma et al., 2011), which allow for mobility (interfragmentary motion) in the fracture gap that stimulates the formation of fracture callus (Epari et al., 2007; Augat et al., 2021; Goodship and Kenwright, 1985; Claes et al., 1995; Lujan et al., 2010; Bottlang et al., 2010). When it comes to intermediate or delayed weight bearing in fracture patients, there is an ongoing debate in the current clinical practice due to fear of fixation failure (Paulsson et al., 2021; Lieder et al., 2021). As a result, the mechanical loading during the earlier phases of bone healing might be limited, which might not be the optimal strategy, as discussed later in this review (Windolf et al., 2021; Glatt et al., 2021). However, in general it must unfortunately be said that potential influences of mechanobiology on the early inflammatory phase of fracture healing currently play no role in clinical decision-making. Only knowledge on mechanics-dependent callus formation is taken into consideration so far.

### 3.2 Mechanics-dependent tissue formation during regeneration: from tissue differentiation theories to mathematical modelling of fracture repair inflammation

As early as 1960, Pauwels proposed the first tissue differentiation paradigm, describing that local hydrostatic pressure transforms MSCs to chondroblasts (cartilage formation) and the shear strain transforms MSCs to osteoblast and fibroblast (bone and fibrous tissue formation) (Pauwels and Pauwels, 1980; Betts and Müller, 2014). Following Pauwels’ pioneering work, several theories and models were developed to describe the progression of fracture healing, considering factors such as cyclic octahedral shear stress, hydrostatic pressure, and blood supply quality (Carter et al., 1988), interfragmentary strain, the combination of interfragmentary strain and pressure (Claes et al., 1995; Claes and Heigele, 1999), and fluid flow and shear strain (Lacroix et al., 2002). The development of these theories and models significantly enhanced our understanding of the impact of local mechanical conditions on healing progression; however, they did not account for the inflammatory mechanisms that follow fracture of a bone. With technological advancements in computational simulations, modeling of cellular and molecular dynamics, as well as inflammatory reactions, has become possible (Lafuen et al., 2021).

The integration of simulation technology with experimental data follows an iterative process that combines computer modeling with empirical data. Borgiani et al. (2023) exemplify such an approach using an agent-based model to simulate immune cells, inflammatory cytokines, fracture debris, and their interactions during the early inflammatory phase of bone healing. Briefly, a model - a mathematical representation of the physical system - is first developed based on parameters derived from prior *in vitro* or *in vivo* studies. The model’s predictions are compared with *in vivo* experimental outcomes (e.g., histological data) to further calibrate its parameters. Subsequently, the model is iteratively updated and refined and finally validated on an independent dataset to evaluate its robustness. Similar approaches have been employed by other groups to investigate cell migration patterns, angiogenesis and different fracture healing phases in small and large animal models (Hedayatzadeh Razavi et al., 2024; Yang et al., 2024; Kendall et al., 2023).

Simulating the early stages of bone healing is challenging for several reasons. The models themselves are complex integrating numerous parameters derived from diverse studies [e.g., cellular migration speed (Segovia-Juarez et al., 2004) and cytokine secretion ratios (Byrne and Reen, 2002)]. Furthermore, obtaining structural data at early healing stages is particularly difficult, as early repair tissue cannot be visualized by computed tomography (CT). This has recently been addressed by atomic force microscopy–based nanoindentation (Hedayatzadeh Razavi et al., 2024). To date, many models of early bone healing have been restricted to two-dimensional representations, where the fracture area serves as the simulation domain (Borgiani et al., 2023; Hedayatzadeh Razavi et al., 2024; Yang et al., 2024), while the role of mechanical loading has been addressed only to a limited extent. However, recent developments in well-controlled animal models may help bridge this gap by enabling the collection of more robust experimental datasets (Wehrle et al., 2021; Hente and Perren, 2021).

Whereas some experimental validation has been reported for simulations of the early healing stage, it remains limited, and thus the predictive power of these models is still constrained [for a review of validation methods of inflammation in fracture healing see (Lafuen et al., 2021)]. Nevertheless, as more high-quality data become available, *in silico* models are expected to become powerful tools for exploring mechano-immunomics in the early healing stage, thereby reducing the number of animals required for *in vivo* experiments and paving the way toward patient-specific models of bone regeneration.

### 3.3 Sensitivity to mechanical cues during different phases of secondary bone healing

To assess the effect of mechanical cues on different phases of fracture healing, several *in vivo* and *in vitro* models have been introduced, which has been reviewed elsewhere [for review see: (Ma et al., 2023)].

#### 3.3.1 Impact of mechanics during early healing stage

Although the post-fracture hematoma and early repair tissue lack significant mechanical competence compared to cartilage or bone (Woloszyk et al., 2022), it has been demonstrated that the early healing stage is highly sensitive to mechanical inputs (Windolf et al., 2021) and that alterations in the local mechanical environment during this stage can influence the healing outcome.

Using an instrumented passive fixation in sheep, Windolf et al. observed a positive correlation between number of loading cycles in the first 2 weeks post-op with the strength of the healed bone (9 weeks post-op), indicating the importance of early stimulation for robust and timely fracture healing (Windolf et al., 2021). Similarly, with an active fixator, Barcik et al. showed that withholding mechanical stimulation for the first 3 weeks delayed healing, whereas applying stimulation from the first postoperative day accelerated the process (Barcik et al., 2023). Moreover, experiments with reverse/inverse dynamization—where high-magnitude stimulation is confined to early healing phase—highlight the advantages of targeting mechanical cues to this specific phase of healing (Glatt et al., 2021; Glatt et al., 2012). Conversely, several *in vivo* experiments indicate that overly flexible fixation can delay the transition from inflammation to the repair phase. Epari et al. found that in sheep treated with more flexible external fixators, remnants of the hematoma persisted for a longer than in more rigidly fixed fractures (Epari et al., 2006). In a mouse model, Sabate Bresco et al. showed that flexible fixators were associated with elevated levels of inflammatory markers, suggesting that excessive instability intensifies local inflammation (Sabate-Bresco et al., 2021).

Hence, although early mechanical loading can be beneficial, excessive flexibility may prolong inflammation and impede timely progression to the repair phase. Furthermore, it remains unclear whether the impact of mechanical cues is consistent throughout the entire duration of inflammation. Using a small animal model, Miclau et al. showed that delaying instrumentation by 24 h (allowing for instability and, thus, large stimulation magnitude) resulted in up to a 40-fold increase in cartilage formation, as observed histologically 10 days post-op. Interestingly, there was no difference in cartilage area between animals treated 24 and 48 h after surgery (Miclau et al., 2007). Taken together, these findings illustrate that mechanical cues play an important role in shaping early repair tissue, with profound consequences for the entire healing process.

#### 3.3.2 Advanced healing stages and remodeling

With the progression of healing and the formation of fracture callus that bridges the fracture gap, interfragmentary strain decreases. Nevertheless, the healing process is still sensitive to mechanical cues. Claes et al. (2011b) showed that late dynamization (from the third and fourth week) in rats resulted in improved healing in comparison to a fixator that was stable throughout the entire 5-week healing period. In a recent study, Wehrle et al. showed the effect of individualized loading on bridged defects in mice. The loading initiated 3 weeks post-op resulted in far more advanced bone formation around the defect compared to 0 N loaded controls (Wehrle et al., 2021). On the other hand, Goodship and Kenwright (1985). Goodship et al. (1998) showed in sheep that applying active stimulation only when the bridging occurs diminished healing progression in comparison to animals stimulated in the early healing stage. In the experiments of Goodship et al. (which were instrumental to our understanding of the importance of stimulation on fracture healing), the same force-controlled loading regime was used for all study groups. This contrasts with the study of Wehrle et al., where loading force was individually computed based on the µCT imaging to ensure favorable stimulatory conditions in the defect (Wehrle et al., 2021). Overall, it suggests that the loading regimes in the advanced healing stages must be carefully tuned to respect the strength of the repair tissue when stimulation is actively applied.

### 3.4 Mechanical regulation of cellular and molecular mechanisms during repair and remodeling

The mechano-responsiveness of cells in the fracture region is regarded as a key factor of biomechanical influences during the fracture healing process and has been shown for many cell types, e.g., MSCs [inflammatory phase; (Josephson and Morgan, 2023)], osteoblasts [repair phase; (Papachristou et al., 2021)], osteoclasts, osteocytes [remodeling phase; (Papachristou et al., 2021)]. Thereby, the mechanotransduction of extracellular stimuli into intracellular biochemical responses mediates gene and protein expression with subsequent effects on the bone healing process [for review of mechanotransduction see: (Vogel, 2006; Vogel and Sheetz, 2006)].

The local mechanical environment changes throughout the healing process, from the hematoma via the formation of the soft and hard callus and subsequent callus remodeling with associated healing phase-specific characteristic gene expression profiles (Hussein et al., 2018). It has to be emphasized, that reported strains within the fracture healing area [e.g., ca. 9′000 µɛ 2 weeks post-op in a rat model (Klosterhoff et al., 2020)] are substantially higher compared to strains measured on intact bone surfaces [ca. 500 - 3′000 µɛ; for review see (Paul et al., 2018)], indicating relevance of the local mechanical environment for the healing process. To link local mechanical properties and gene expression in the healing area early after fracture, Woloszyk et al. (2022) used a femur defect model in rats with different defect sizes to mimic normal (0.5 mm), delayed (1.5 mm) and non-healing (5 mm) bone defects. Bulk RNA sequencing and scanning electron microscopy (SEM) of the hematoma at post-operative day 3 in subsets of animals revealed distinct gene signatures and structural properties of the hematoma in the different healing conditions. Novel multimodal mechanically-controlled femur defect models in mice have enabled to link local mechanical callus properties [e.g., effective strain; (Tourolle Ne Betts et al., 2020; Paul et al., 2022)] with histology-based gene and protein analyses (Wehrle et al., 2021). Specifically, using a recently established spatial transcriptomics protocol for formalin-fixed paraffin-embedded (FFPE) musculoskeletal tissue samples from mice (Wehrle et al., 2024), Mathavan et al. were able to link the transcriptomic responses of cells to the local strain magnitude within the defect region during the remodeling phase of fracture healing (Mathavan et al., 2024). Looking further downstream, multiple signaling pathways (e.g., Estrogen, Wnt, BMP) have been characterized as mechano-responsive with implications for bone biology and fracture healing [for review see: (Wang et al., 2022; da Silva Madaleno et al., 2020)]. Recently, mechanosensitive Piezo channels as well as the transcriptional coactivators yes-associated protein 1 (YAP) and transcriptional coactivator with PDZ-binding motif (TAZ) turned in the focus. Chen et al. (2021) showed that YAP/TAZ deletion of endothelial Piezo1 resulted in impaired bone healing with inhibition of osteoblast maturation and ossification. Via constitutive and developmental YAP/TAZ deletions in Osterix-expressing cells in mice, Kegelman et al. (2021) comprehensively assessed and characterized that YAP and TAZ promote periosteal osteoblast precursor expansion and differentiation for fracture repair.

Based on the importance of the local mechanical conditions in the bone healing region, many *in vivo* studies have applied and assessed external mechanical loading as a treatment option for (healing phase-specific) improvement of impaired bone healing conditions [reviewed in (Ma et al., 2023); low-magnitude high-frequency vibration: (Steppe et al., 2020), exercise: (Song, 2022)]. In combination with targeted transgenic mouse models, precisely controlled mechanical regimens have enabled wider cellular and molecular understanding of the local microenvironment and mechanics in respect to angiogenesis, angio-osteogenic coupling and chondro-osteogenic trans-differentiation relevant for successful bone healing (Wazzani et al., 2021; Wong et al., 2018). Coupling of angiogenesis and osteogenesis has been described for loadbearing bones, whereas a recent study by Bixel et al. showed that angiogenesis is uncoupled from osteogenesis during non-loadbearing calvarial bone regeneration (Bixel et al., 2024), indicating a strong influence of the local mechanical conditions on this mechanism during bone healing. In addition, *in vitro* setups and models have allowed for further characterizing the effect of specific mechanical protocols and parameters (e.g., fluid flow stress, compressive and tensile strain) on specific cells involved in the different healing phases of fracture repair including the assessment of lineage commitment, differentiation and gene/protein expression profiles [reviewed in (Wu et al., 2024)]. Such targeted systems have allowed for characterizing the mechano-responsiveness of specific cell types (e.g., MSCs, osteoblasts, osteoclasts, osteoclasts) involved in bone healing, however they often resemble a simplified setup (one type of mechanical stimulus, 2D vs. 3D, selected molecular markers as readout) and lack the possibility for capturing and monitoring of the local mechanical parameters in the system. To mechanistically study fluid-flow dependent lineage commitment of MSCs, Song et al. (2012) developed a platform based on confocal imaging of cell surface bound-fluorescent microbeads enabling for measuring cell surface strain in live cells in response to controlled delivery of stresses. Recent developments have focused on *in vitro* platforms enabling to study synergistic effects of multiple externally applied cues [e.g., combined mechanical cues, combined biochemical and physical cues; (Josephson and Morgan, 2023; Jesus et al., 2022)]. Further developments are targeted towards specific *in vitro* platforms to study aspects of bone healing. Whelan et al. (2023) developed a micro-physiological model integrating endothelial cells and organoids mimicking different stages of endochondral bone development within a microfluidic chip. Via combining such *in vitro* systems with longitudinal monitoring of mechanical parameters in the microenvironment and larger scale molecular readouts as well as computational analyses, they may also provide a basis for microenvironment-targeted treatment strategies for bone healing (Hao et al., 2023; Zhu et al., 2021). Several other devices have been used to simulate the effect of mechanical loading/presence of mechanical stimuli during fracture healing *in vivo* and *in vitro*. *In vitro*, various bioreactors systems have been used to apply compression or shear stress to the cells, likewise shear stress may be delivered via vibration platforms with attached cell culture plates and cyclic tension (strain) may be simulated via the commercially available FlexCell tension system or four-point bending devices [reviewed in: (Michael Delaine-Smith et al., 2015; Ladner et al., 2022)].

## 4 “Mechanobiology reloaded”: interplay between mechanics and inflammation during fracture healing

### 4.1 Influence of hematoma stiffness and biomechanical stimulation on inflammation

During early fracture healing, the formation of the fibrin-rich hematoma induces strong changes in the tissue mechanical environment which exert various forms of biomechanical stimulation (e.g., changes in the tissue stiffness, local shear stress conditions, compression loading and mechanical stretch) on immune cells.

Platelets are the first immune cells to be recruited from the circulation to the site of vascular injury after the fracture. Platelet adhesion to the vascular endothelium, aggregation and activation play a crucial role, together with the coagulation cascade, in blood clot formation (Baht et al., 2018; Oshinowo et al., 2023). Activated platelets secrete pro-inflammatory cytokines to recruit other immune cells (neutrophils and monocytes) and mesenchymal progenitors to the site of injury (Baht et al., 2018). Platelets sense and respond to biomechanical stimuli such as local shear stress conditions and changes in substrate stiffness (Oshinowo et al., 2023) and these modulate platelet adhesion, aggregation and activation (Woloszyk et al., 2022; Nesbitt et al., 2009; Kulkarni et al., 2000; Shi et al., 2016; Mor et al., 2022; Qiu et al., 2014). Nesbitt et al. showed that discoid platelets were found to form stable aggregates *in vivo* at sites of rapid changes in blood flow such as vascular injury, inducing micro-gradients in shear stress (Nesbitt et al., 2009). Moreover, Morell et al. subjected human platelets to laminar and turbulent flow in an *in vitro* flow-and-cone system for 120 min and observed an increase in platelet activation by laminar flow, and even more so by turbulent flow (Mor et al., 2022). Platelets within the developing fibrin-rich blood clot may also mechano-sense changes in substrate stiffness as coagulation factors such as thrombin induced the conversion of fibrinogen to fibrin (Oshinowo et al., 2023). Qui et al. observed a stiffness-dependent increase in human platelet adhesion and spreading on fibrinogen immobilized polyacrylamide (PAA) gels ranging from 0.25–50 kPa *in vitro*, and higher platelet activation on gels stiffer than 5 kPa (Qiu et al., 2014). Platelet activation plays a crucial role for remodeling of the fibrin-rich matrix and strengthening of the hematoma as platelets contract and thereby bend and shorten fibrin fibers via their filopodia (Lam et al., 2011; Kim et al., 2017).

Likewise, neutrophil adhesion to the vasculature, transmigration and chemotaxis as well as the activation in response to injury have been shown to be sensitive to the stiffness of the underlying substrate (Oakes et al., 2009; Jannat et al., 2010; Abaricia et al., 2021). Human neutrophils spread more on PAA hydrogels with moduli of 12 kPa and demonstrated directed migration towards a chemotactic gradient as compared to on gels with a stiffness of 0.3 kPa (Jannat et al., 2010). Moreover, hematoma stiffness may control neutrophil pro-inflammatory behavior as Abaricia et al. showed enhanced NET formation and IL-1ß-, TNF-α-, MCP-1-, MIP-1α-, CCL5-, CXCL1 secretion from mouse neutrophils on polydimethylsiloxane substrates with increasing stiffness (0.2–32 kPa) (Abaricia et al., 2021).

This increase in pro-inflammatory cytokines at the fracture site may lead to an enhanced recruitment of circulating monocytes into the hematoma (Baht et al., 2018). Monocytes and monocyte-derived macrophages are found at the site of tissue injury and play a crucial role in the repair and remodeling of tissues subjected to mechanical stress (Ar et al., 2007; Blomgran et al., 2016; Bergmann et al., 2006; Krieger et al., 2016; Schlundt et al., 2018; Raggatt et al., 2014). Fahy et al. investigated the effect of shear and compression on monocytes entrapped in alginate gels via a multiaxial loading bioreactor. Loading for 1 h/day for 3 consecutive days increased primary human monocyte IL-6 and IL-8 gene expression levels compared to free swelling controls and the protein levels of TNF-α, MIP-1α and IL-13 (Fahy et al., 2019). Shear and compression loading may exert differential effects on the inflammatory milieu during endochondral fracture healing. Fahy et al. found that compression loading alone enhanced the production of IL-1ß from the human monocyte reporter cell line THP-1, as well as the expression of inducible nitric oxide synthase (NOS2), which is expressed in the fracture callus during the initial stage of repair (Diwan et al., 2010), while shear increased MCP-1 secretion (Fahy et al., 2019). Mechanical stimuli may also influence monocyte to macrophage differentiation as Yang et al. found that applying 4% strain at 1 Hz for 24 h to human monocytes increased the monocyte differentiation-associated transcription factor PU.1 (Yang et al., 2000). Evidence suggests that macrophages have a profound impact on fracture healing. Recruitment of macrophages to the fracture site is crucial for vascularization, callus formation, cartilage maturation and callus remodelling (Xing et al., 2010). In addition, a reduced number of macrophages during fracture healing in murine femoral defect models has been shown to impair endochondral ossification and overall delay fracture healing (Schlundt et al., 2018; Raggatt et al., 2014). The presence of pro-inflammatory M1 macrophages and their secreted factors in conditioned medium have been reported to enhance the osteogenesis of bone marrow-derived MSCs (Guihard et al., 2012; Lu et al., 2017). Moreover, enhancing the M2 macrophage phenotype *in vivo* improved early bone regeneration (Schlundt et al., 2018). It has been recognized that besides soluble factors, biomechanical cues influence macrophage phenotype and function (McWhorter et al., 2015; Escolano et al., 2021). Strain and vibration treatment have been shown to alter macrophage polarization, cytokine secretion and ECM remodeling. Cyclic strain has been found to modulate macrophage phenotype in three-dimensional (3D) scaffolds, depending on the strain rate. While strain magnitudes of 7%–8% at a frequency of 0.8 Hz drive human peripheral blood-derived monocyte polarization towards an M2 phenotype over 7 days, in line with an increase in the secretion of anti-inflammatory cytokines like IL-10 and TGF-ß1, higher strains (12%–14%) induce an M1 phenotype concomitant with enhanced IL-6, TNF-α and IL-1ß levels (Ballotta et al., 2014; Bonito et al., 2018). Contrary, Tu et al. found an increased IL-1ß and IL-6 secretion from murine macrophages with increasing strain amplitudes (5%, 10%, 15%) after 8h, as well as in both the M1 and M2 phenotype (Tu et al., 2022). Species-dependent differences in macrophage pro-inflammatory response as well as differences arising from the length and time-scale of the observations have been reported before (McWhorter et al., 2015). Macrophages are known to contribute to the secretion of ECM components to promote new tissue formation by fibroblasts and are also capable of ECM remodeling (Weitkamp et al., 1999; Schnoor et al., 2008; Werb et al., 1980). Applying strain was found to have little influence on the production of ECM components by human peripheral blood monocyte-derived macrophages (Ballotta et al., 2014; Bonito et al., 2018), however, 4% strain was found to upregulate MMP1 and 3 expression (Yang et al., 2000). Low magnitude high frequency vibration (35 Hz, 0.3 x g; 20 min/day 5 days/week) increased the recruitment of macrophages to the fracture site in ovariectomy-induced osteoporotic rats, enhanced M1/M2 transition, IL-6 and TNFα expression and reduced IL-10 levels 1 week post-operatively (Chow et al., 2019). On the other hand, Pongkitwitoon et al. observed that vibration treatment (100 Hz, 0.15 x g; 20 min/2x/day 2 h break) of murine macrophages *in vitro* reduced protein levels of INF-γ, IL-6 and TNFα after 1 and 3 days of stimulation while enhancing proliferation (Pongkitwitoon et al., 2016). A large amount of studies investigated the effect of substrate stiffness on macrophage recruitment (Blakney et al., 2012; Ni et al., 2023), morphology (Escolano et al., 2021), migration mode (Sridharan et al., 2019), phagocytosis (Scheraga et al., 2016; Patel et al., 2012), phenotype and cytokine production (Blakney et al., 2012; Ni et al., 2023; Chen et al., 2020; Okamoto et al., 2018). In general, bone marrow-, RAW 264.7- and THP-1-derived macrophages cultured on PAA hydrogels with various coatings possess a more round morphology and secrete higher levels of M1 markers (IL-1ß, TNF-α) with lower stiffness moduli (0.2–1 kPa), while stiffer gels (16–150 kPa) induce a spread morphology and higher levels of M2 markers (Escolano et al., 2021; Scheraga et al., 2016; Patel et al., 2012; Chen et al., 2020; Carnicer-Lombarte et al., 2019; Xing et al., 2021). However, others have observed a more pro-inflammatory macrophage phenotype on stiffer gels ranging from 840–230 kPa (Blakney et al., 2012; Sridharan et al., 2019; Previtera and Sengupta, 2016). These differences may arise from the use of different ligands, coating densities or factors to induce inflammation (Escolano et al., 2021). Also, mechanostimulation protocols in term of loading, frequencies, amplitudes and duration differ a lot, potentially leading to different results. Furthermore, as mentioned earlier, species-differences might have influenced the contrasting results reported in these studies. Another important factor to consider would be a specific time-dependency of mechanobiological responses of immune cells, which is very likely due to the highly dynamic nature of immune cell responses.

The stiffness of the underlying substrate has also been shown to modulate T-cell activity. Majedi et al. cultured T-cells on microporous, alginate-based, RGD-functionalized 3D scaffolds with stiffness moduli of 4 and 40 kPa. They observed faster T-cell migration, higher proliferation and IL-2, IFN-γ and TNF-α expression as well as an upregulation of the T-cell activation surface marker CD25 on the stiffer scaffolds (Majedi et al., 2020).

These findings imply that mechanical cues in the tissue microenvironment may mediate cellular responses during the inflammatory phase of fracture healing. However, variations in the duration and magnitude of the stimuli, the time period of observation and the presence/absence of various factors to induce inflammation and species-dependent differences (in particular between studies using cells from murine- and human origin) make it difficult to transfer this knowledge to improve fracture healing in the clinical setting. Another concern is that most of the findings are currently based on *in vitro* studies. *In vivo* studies identifying which mechanical stimuli are relevant to consider during fracture healing and whether these stimuli are determinant of cell behaviour *in vivo* are currently underrepresented. However, measuring local tissue strains or fluid shear stresses induced by e.g., fixator stiffness or gap size and linking these stimuli to cellular responses and tissue formation during early fracture healing *in vivo*, is challenging (Duda et al., 2023).

### 4.2 Influence of mechanical stability on the inflammatory phase

The first hints that mechanical stability is influencing the inflammatory phase of fracture healing were given by the extensive work of Duda and colleagues [reviewed in (Schmidt-Bleek et al., 2014) and (Knecht et al., 2021)]. In 2012, they compared the early and late healing phases in an uneventful fracture healing scenario versus a mechanically induced delayed healing model in sheep (Schmidt-Bleek et al., 2012). In the early phase, they found significantly higher T-lymphocyte counts in the mechanically delayed model, both in the hematoma itself and the adjacent bone marrow. Especially cytotoxic T-cells were highly present. Furthermore, the periosteum at the early healing phases showed lower expression of hematopoietic stem cell markers and angiogenic factors, indicating that the unfavorable mechanical environment directly interferes with inflammation and therefore also regeneration (Kolar et al., 2010). In 2014, Ode et al. did an unbiased whole genome expression analysis of fracture hematoma tissue under different mechanical conditions (Ode et al., 2014). They found more than 1000 differentially regulated genes, with 144 genes being regulated by both age and fixation stability (which is interesting because age is proposed as a regulator of bone mechanosensitivity itself). Functional annotation analyses revealed an interplay between mechanics, inflammatory genes and genes which influence later repair stages, like ECM modulating matrix metalloproteinases (MMPs). In 2024, Mehl et al. detected that extrinsically imposed shear stress in the gap delayed hematoma remodeling and shaped the morphology of early collagen fiber orientations and microvascular networks, suggesting that enhanced shear increased the nutrient exchange between cells in the hematoma (Mehl et al., 2024). All these data implies that fracture fixation stability is able to modulate very early healing responses during bone regeneration and that this interferes with the later repair response. Conversely, that would mean that clinical fixation devices should not only be optimized towards their biomechanical properties for later healing stages, but also towards the early healing stages (as already mentioned in chapter 3.3). Eventually, this might lead to the need for adjustable devices as described for small and large animal models (Glatt et al., 2021; Muller et al., 2015).

When reviewing the influence of mechanical stability on fracture healing, it would be interesting to compare the early healing phases in long-bone fracture models vs. non-locomotion loaded bones like the skull bone. However, also cranial bones are loaded due to muscle forces and it is not really known in small animals how the magnitudes of strains are different between for example, a mandible healing model or a long bone healing model. Therefore, it is challenging to draw specific conclusions for mechano-immunomics in those models.

A great barrier to progress in the field and gain a better understanding of the effect of the mechanics during the inflammatory phase of fracture healing currently represents the spareness of robust and reliable *in silico* models, in particular models which combine computer modeling with data from experimental studies (Lafuen et al., 2021). The development of such novel *in silico* models should focus both on modeling the biological factors such as cells/cell densities and the temporal/spatial distributing of pro- and anti-inflammatory agents present during the inflammatory phase (Kojouharov et al., 2017. Modeling the effects of inflammation in fracture healing; Trejo et al., 2019. Modeling the macrophage-mediated inflammation involved in bone fracture healing. Process. Math. Comput. Appl. 24, 12), as well as the mechanobiology, and determine the effect of these combined initial factors on the fracture healing outcome (Mathavan et al., 2025; Ghiasi et al., 2019). These models would allow to better map local strains in the fracture area with cellular reactions, therefore leading to better conclusions regarding mechano-immunomic processes.

### 4.3 Molecular mechanisms: mechanosensation of immune cells

The basis for the development of the mechano-immunomics field are that there is a deeper understating of mechanotransduction in immune cells. Several mechanosensors have been identified in immune cells, among them the mechanosensitive calcium (II)-ion (Ca^2+^)-permeable channels Piezo1 (Hamza et al., 2021; Solis et al., 2019; Atcha et al., 2021; Baratchi et al., 2020) and transient receptor potential vanilloid-type 4 (TRPV4) [(Michalick and Kuebler, 2020)] as well as the transcriptional co-activator YAP (Meng et al., 2020; Meli et al., 2020).

Piezo1 controlled Ca^2+^-influx upon mechanical stimulation plays a crucial role in the regulation of Ca^2+^-dependent signaling pathways in immune cells, modulating effector functions (Du et al., 2023). E.g., Piezo1-dependent Ca^2+^-influx mediated the shear-stress induced activation of human monocytes, leading to enhanced monocyte adhesion, phagocytosis and pro-inflammatory behavior (Baratchi et al., 2020), as well as activation and cytokine expression of primary human T-cells (Hope et al., 2022). Moreover, Piezo1 has been identified as mechanosensor of substrate stiffness in bone marrow-derived macrophages as Atcha et al. observed higher Piezo1 expression and Ca^2+^-activity of macrophages on stiffer PAA hydrogels (280 kPa) compared to soft ones (1 kPa)associated with increased *NOS2* gene expression and regulated via the cytoskeleton (Atcha et al., 2021). However, evidence suggests that Piezo1 may also contribute to anti-inflammatory effects as murine macrophages demonstrated reduced pro-inflammatory gene expression, M2 polarization and TGF-ß1- secretion, stimulation MSC osteogenesis (Hamza et al., 2021).

Moreover, Yin et al. demonstrated that TRPV4 is a major regulator of neutrophil function as its deficiency impaired the production of reactive oxygen species, adhesion, and transmigration of murine neutrophils in response to pro-inflammatory stimuli (Yin et al., 2016). Dutta et al. demonstrated the crucial role of TRPV4 in mediating substrate stiffness-induced M1 macrophage polarization, as TRPV4 ablation *in vivo* and *in vitro* lead to reduced upregulation of M1 markers in response to increased substrate stiffness compared to wild-type (Dutta et al., 2020).

Mechanosensing of substrate stiffness by immune cells is also conveyed through transcriptional co-activator YAP and has been shown to modulate T-cell- and macrophage activity (Meng et al., 2020; Meli et al., 2020). Meng et al. demonstrated that YAP mediates the nuclear localization of the transcription factor nuclear factor of activated T-cells 1 (NFAT1) on stiff substrates and inhibits translocation on compliant substrates, thereby controlling T-cell proliferation and metabolic activity (Meng et al., 2020). Moreover, Meli et al. observed a stiffness-dependent increase in the pro-inflammatory response of human monocyte-derived macrophages in response to nuclear translocation of YAP (Meli et al., 2020).

Mechanosensation of immune cells is also conveyed via integrins and the cytoskeleton (Du et al., 2023; Ross et al., 2013). Stiffness-dependent effects on neutrophil-, and macrophage pro-inflammatory behavior and T-cell function have been shown to be mediated by integrin/focal adhesion kinase (FAK) signaling (Abaricia et al., 2021; Majedi et al., 2020; Hsieh et al., 2019) and actomyosin contractility (Escolano et al., 2021).

**FIGURE 1:**
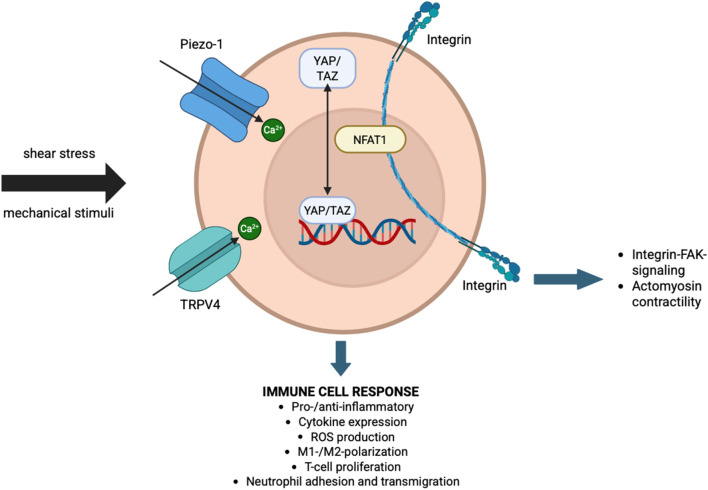
Interaction of mechanotransduction and immune response. Schematic representation of key mechanosensors and downstream pathways through which immune cells respond to mechanical stimuli such as shear stress. Mechanical inputs are sensed by Piezo-1 and TRPV4 ion channels, leading to Ca (Hellwinkel et al., 2020)+ influx and activation of transcriptional regulators such as YAP/TAZ and NFAT1. Integrins further mediate mechanotransduction via FAK signaling and actomyosin contractility. These pathways converge to regulate immune cell behavior, including pro- or anti-inflammatory responses, cytokine expression, reactive oxygen species (ROS) production, macrophage polarization (M1/M2), T-cell proliferation, and neutrophil adhesion and transmigration.

In summary, clear evidence identifies the role of mechanosensation by immune cells in modulating inflammatory responses, however, up to date, the precise molecular mechanisms on how mechanotransduction modulates cellular responses remains unclear. This is mainly due to the fact that current *in vitro* and *in vivo* studies solely focus on target approaches, e.g., knockout studies to inhibit or lose mechanosensory activity. Untargeted ‘global’ approaches including novel omics technologies such as proteomics or transcriptomics would help to gain a better understanding of downstream effects by e.g., identifying the modulators and interaction partners of mechanosensors. A great example of a novel technique which could be used is the combination of finite element modeling, *in vivo* µCT imaging and spatial transcriptomics, as recently introduced by Mathavan et al. (Mathavan et al., 2025). A summary of mechanically induced pathways in immune cells, which might be relevant during fracture healing, is depicted in Figure 1.

## 5 Clinical perspective

While the bulk of evidence for mechanical modulation of fracture healing originates from preclinical models, emerging translational and clinical data provide encouraging support for these concepts in humans. A completed multi-center observational study of distal femur fractures treated with far-cortical locking screws demonstrated the feasibility and safety of inducing controlled axial micro-motion via reduced implant stiffness to actively promote symmetric callus formation (Braun et al., 2021). Moreover, a randomized clinical investigation involving 80 tibial fracture patients stabilized with external fixators and subjected to daily interfragmentary micromovement (1 mm at 0.5 Hz for 20 min) showed a statistically significantly faster attainment of healing, defined by bending stiffness, compared to patients in the non-stimulated control group (23 weeks vs. 29 weeks on average) (LE, 2022). Additionally, narrative reviews highlight clinical interventions such as axial micromovement protocols, electromagnetic stimulation, and low-intensity pulsed ultrasound, that have demonstrated up to 20%–30% reductions in healing time in human patients when properly applied (Ganse, 2024). While these findings are promising, the limited number of trials and variability in methodologies underscore the need for more rigorous, phase-specific clinical studies. Future trials should aim to establish optimal loading parameters, timing, and safety profiles to translate mechanobiological insights into reliable therapeutic strategies. Also, so far the clinical trials do not really take the concept of mechano-immunomics into concept which should be considered in the future.

## 6 Conclusion

**FIGURE 2:**
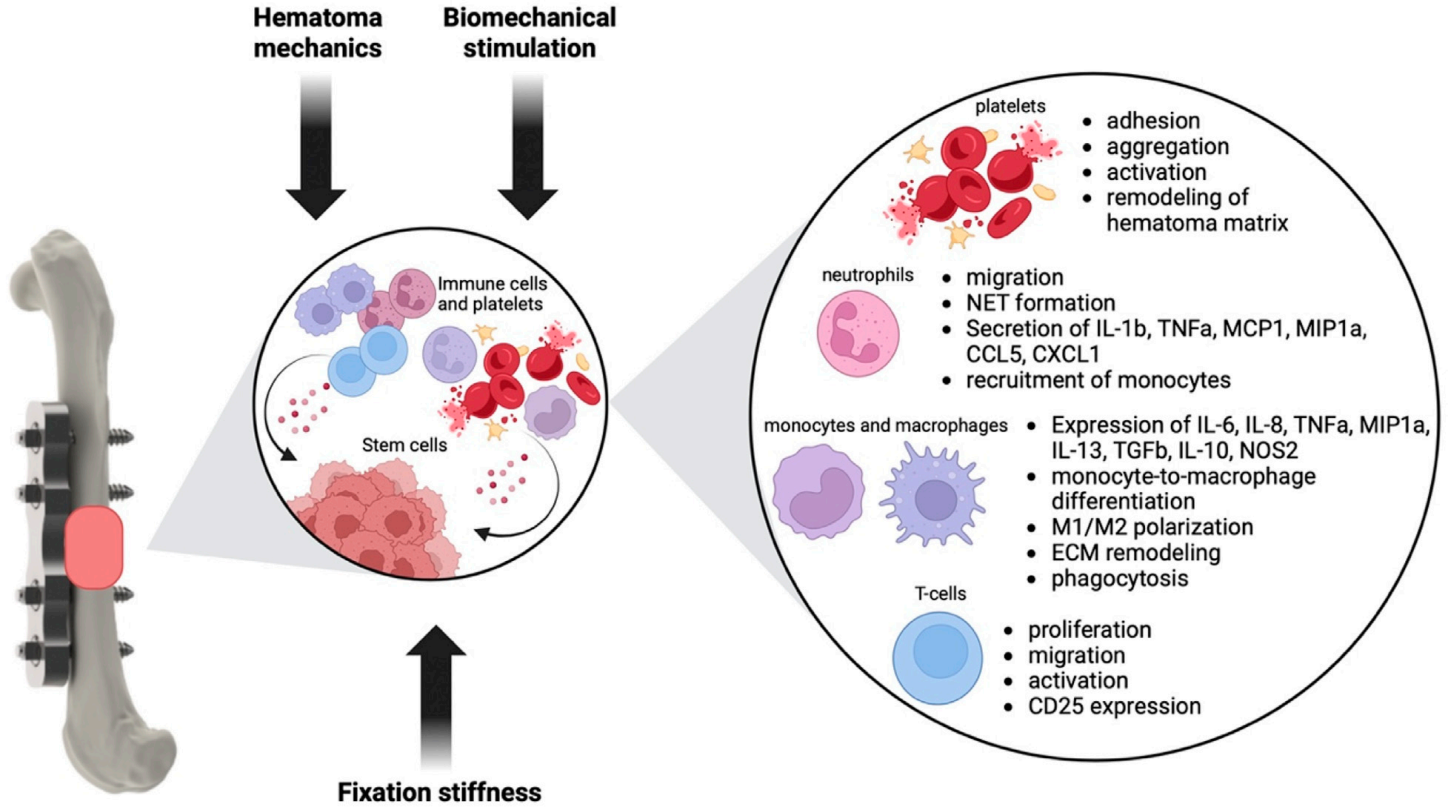
Influence of mechanical cues on the inflammatory phase of fracture healing. Mechanical cues like fixation stiffness and extend of loading, the hematoma mechanics and biomechanical stimulation might influence inflammatory cells like neutrophils, monocytes, macrophages and T-cells as well as platelets. Changes in the phenotype and behavior of these cell types in turn will influence the hematoma environment and the regenerative potential of stem cells, thereby shaping later healing stages (Baht et al., 2018; Woloszyk et al., 2022; Oshinowo et al., 2023; Nesbitt et al., 2009; Kulkarni et al., 2000; Shi et al., 2016; Mor et al., 2022; Qiu et al., 2014; Oakes et al., 2009; Jannat et al., 2010; Abaricia et al., 2021; Ar et al., 2007; Blomgran et al., 2016; Bergmann et al., 2006; Krieger et al., 2016; Schlundt et al., 2018; Raggatt et al., 2014; Fahy et al., 2019; Yang et al., 2000; Guihard et al., 2012; Lu et al., 2017; McWhorter et al., 2015; Escolano et al., 2021; Blakney et al., 2012; Ni et al., 2023; Sridharan et al., 2019; Scheraga et al., 2016; Patel et al., 2012; Chen et al., 2020; Okamoto et al., 2018; Majedi et al., 2020).

Decades of basic, translational and clinical research has investigated the influence of mechanics on the repair and remodeling phase of fracture healing. The basic tissue differentiation hypothesis of how mechanical cues shape the fracture callus development was already postulated by Pauwels in 1960. Since then, this hypothesis was further refined and developed based on more sophisticated approaches of combining *in silico* modeling and *in vivo* data. Clinical fracture fixation devices have been and are still being developed based on these data to allow successful bone regeneration. However, although we know a lot about the general influence of mechanics on the fracture healing process, still 5%–10% of all fracture patients suffer from delayed healing or non-union formation. Part of these numbers will be based on insufficient biology, but the other part might also be attributed to an incomplete understanding of the mechanobiology of fracture healing. It is also well established that the early inflammatory phase of fracture healing might be decisive for the later regeneration process by inducing angiogenesis, stem cell invasion and cartilage and bone tissue formation. And while traditionally, biomechanical orchestration and inflammation were viewed as distinct phenomena, recent research has illuminated the intricate relationship between mechanics and inflammation in the mechanobiology of fracture healing. In this review, we identified the following key features of how mechanics influences inflammation during fracture healing (Figure 2).

• Fixation stiffness alters local strains, thereby influencing T cell migration towards the fracture hematoma and guides early gene expression of ECM modulating genes
• The mechanical environment in the early fracture hematoma might influence platelet adhesion and activation; neutrophil adhesion, transmigration, chemotaxis, and inflammatory phenotype; recruitment of monocytes; differentiation and polarization of macrophages; and T-cell migration, proliferation and activity.
• External biomechanical stimulation is able to induce changes in immune cell numbers during the early healing stages.
• Known mechanotransduction pathways in immune cells are Piezo channels, TRPV4 as well as the transcriptional co-activator YAP

This leads us to the direction that a more sophisticated approach with a guided “mechano-immuno-therapy” might help to ensure proper fracture healing in patients. This means that when designing fracture fixation implants, not only “conventional” biomechanical considerations regarding callus tissue differentiation should be taken into account, but also information about how mechanics influence inflammation and even the inflammatory status of the patient. For this approach, one would need to determine the optimal mechanical environment to guide inflammation towards regeneration (a topic which is clearly under-investigated so far) and taking into consideration the knowledge we have about mechanobiology in later healing stages. As mechanical requirements might change during the fracture healing process, this also showcases the need for adjustable implants dependent on the stage of fracture healing. From a clinical perspective, in conclusion our findings underscore that implant design should not only focus on ensuring mechanical stability for callus formation, but also on modulating the inflammatory response during the early healing phase. For example, fixation stiffness directly influences immune cell recruitment and cytokine expression within the fracture hematoma, thereby shaping the transition from inflammation to repair. Excessive instability has been associated with prolonged inflammation and impaired angiogenesis, while an overly rigid construct may suppress beneficial mechanosensitive immune responses. This knowledge points toward the development of adaptive fixation systems such as dynamically adjustable plates, external fixators, or splints that can provide stage-specific mechanical environments: initially promoting a balanced inflammatory response, and later enhancing callus maturation. In this way, implant concepts that integrate the concept of “mechano-immuno-therapy” could open new avenues for improving clinical outcomes, particularly in patients at risk for delayed healing or the development of non-unions. However, it is also important to acknowledge that while mechanical modulation strategies hold promise, potential risks must also be considered. Excessive loading during the early inflammatory phase can prolong inflammation, impair vascularization, and delay the transition into the reparative stage, ultimately predisposing to delayed union or non-union. Similarly, fixation constructs that are too flexible may generate persistent micromotion, sustaining high levels of pro-inflammatory cytokines and disrupting hematoma remodeling. On the other hand, overly rigid fixation may suppress beneficial mechanosensitive immune responses and limit callus formation. These findings emphasize the need for a careful, phase-specific balance: early controlled mechanical stimulation appears advantageous, but both under- and over-stimulation can be detrimental. For clinical translation, as mentioned above, adjustable fixation systems and tailored loading protocols should therefore be developed with safeguards to avoid adverse outcomes, ensuring that the therapeutic benefit outweighs the potential harm.

Barriers to investigate the mentioned areas in more detail are clearly that highly interdisciplinary teams are needed for that kind of research, involving cell biologists, immunologists, clinicians, engineers and bioinformaticians. Another barrier which lies in the nature of highly interdisciplinary teams is, that a common language needs to be found for all these disciplines. Also, there are technological barriers, e.g., the lack of adequate *in vitro* and *in silico* models to study mechano-immunomics and the complexity of existing *in vivo* models. Novel techniques like high-resolution spatial transcriptomics might be utilized to overcome methodological issues. Furthermore, another barrier for translation might be species differences, which are also clearly under-investigated in that area. To overcome these barriers would be needed to get further mechanistic insight into the field of mechano-immunomics during fracture healing.
